# β-K(VO_2_)_2_(PO_4_)

**DOI:** 10.1107/S1600536812049884

**Published:** 2012-12-12

**Authors:** Safa Ezzine Yahmed, Meriem Ayed, Mohamed Faouzi Zid, Ahmed Driss

**Affiliations:** a Laboratoire de Matériaux et Cristallochimie, Faculté des Sciences de Tunis, Université de Tunis ElManar, 2092 Manar II Tunis, Tunisia

## Abstract

A new vanadium oxide, potassium bis­(dioxovanad­yl) phosphate, β-K(VO_2_)_2_(PO_4_), has been synthesized by a solid-state reaction. In the title compound, the [V_2_PO_8_] framework is built up from infinite pyramidal [V_2_O_8_]_∞_ and [VPO_7_]_∞_ chains linked together by V—O—P bridges, leading to a three-dimensional framework which delimits two types of inter­secting tunnels running along [100] and [010] in which the four unique K^+^ ions, showing coordination numbers of nine and ten, are located.

## Related literature

For α-K(VO_2_)_2_(PO_4_), see: Berrah *et al.* (1999 ▶). For background to the physico-chemical properties of related compounds, see: Daidouh *et al.* (1997 ▶); Pierini & Lombardo (2005 ▶). For details of structurally related compounds, see: Leclaire & Raveau (2006 ▶); Amoros & Le Bail (1992 ▶); Lii & Wang (1989 ▶); Daidouh *et al.* (1998 ▶); Benhamada *et al.* (1991 ▶). For the preparation, see: Ezzine *et al.* (2009 ▶). For bond-valence parameters, see: Brown & Altermatt (1985 ▶).

## Experimental

### 

#### Crystal data

K(VO_2_)_2_(PO_4_)
*M* *_r_* = 299.95
Triclinic, 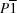

*a* = 4.7438 (8) Å
*b* = 13.889 (2) Å
*c* = 21.201 (3) Å
α = 70.89 (2)°
β = 89.55 (3)°
γ = 88.66 (3)°
*V* = 1319.5 (4) Å^3^
*Z* = 8
Mo *K*α radiation
μ = 3.71 mm^−1^
*T* = 298 K
0.28 × 0.18 × 0.12 mm

#### Data collection

Enraf–Nonius CAD-4 diffractometer
Absorption correction: ψ scan (North *et al.*, 1968 ▶) *T*_min_ = 0.447, *T*_max_ = 0.668
7567 measured reflections
5673 independent reflections
4828 reflections with *I* > 2σ(*I*)
*R*_int_ = 0.021
2 standard reflections every 120 min intensity decay: 1.2%

#### Refinement

*R*[*F* ^2^ > 2σ(*F* ^2^)] = 0.030
*wR*(*F* ^2^) = 0.086
*S* = 1.08
5673 reflections
434 parameters
Δρ_max_ = 0.80 e Å^−3^
Δρ_min_ = −0.50 e Å^−3^

### 

Data collection: *CAD-4 EXPRESS* (Duisenberg, 1992 ▶; Macíček & Yordanov, 1992 ▶); cell refinement: *CAD-4 EXPRESS*; data reduction: *XCAD4* (Harms & Wocadlo, 1995 ▶); program(s) used to solve structure: *SHELXS97* (Sheldrick, 2008 ▶); program(s) used to refine structure: *SHELXL97* (Sheldrick, 2008 ▶); molecular graphics: *DIAMOND* (Brandenburg, 1998 ▶); software used to prepare material for publication: *WinGX* (Farrugia, 2012 ▶).

## Supplementary Material

Crystal structure: contains datablock(s) I, global. DOI: 10.1107/S1600536812049884/fi2128sup1.cif

Structure factors: contains datablock(s) I. DOI: 10.1107/S1600536812049884/fi2128Isup2.hkl

Additional supplementary materials:  crystallographic information; 3D view; checkCIF report

## Figures and Tables

**Fig. 1.**
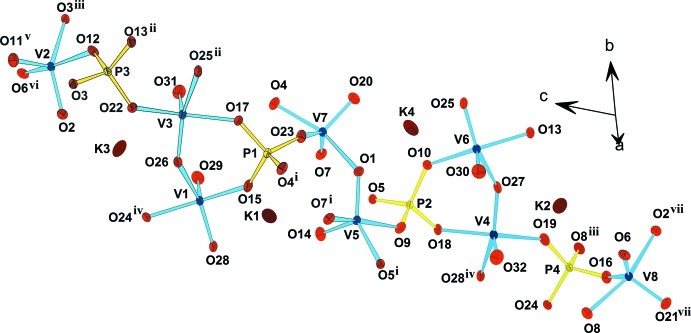
Asymmetric unit of β-K(VO2)2(PO4). Displacement ellipsoids are drawn at the 50% probability level. Symmetry codes:(i) x+1, y, z; (ii) - x+1, -y+2, -z+1; (iii) x-1, y, z; (iv) -x+1, -y+1, -z+1; (v) x, y, z+1; (vi) x-1, y, z+1; (vii) x, y, z-1.

**Fig. 2.**
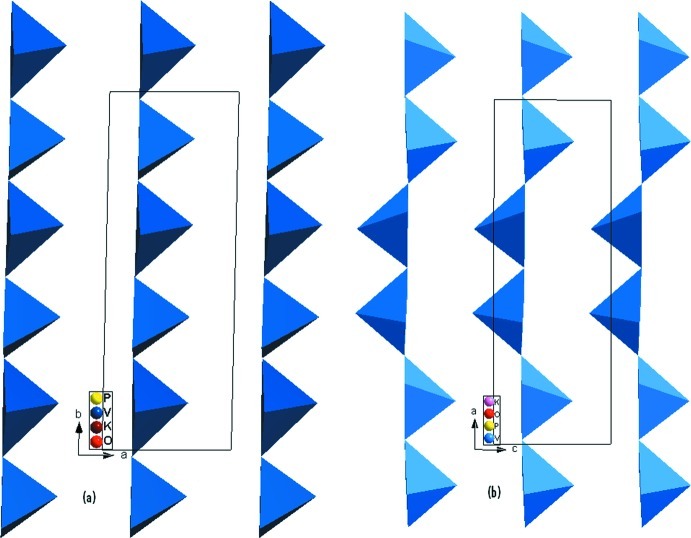
Representation of the first type of infinite chains (V2O8)∞ showing the disposition of apical oxygen atoms: (a) in cis-cis position in β-K(VO2)2(PO4). (b) in cis-cis-trans-trans position in α-K(VO2)2(PO4).

**Fig. 3.**
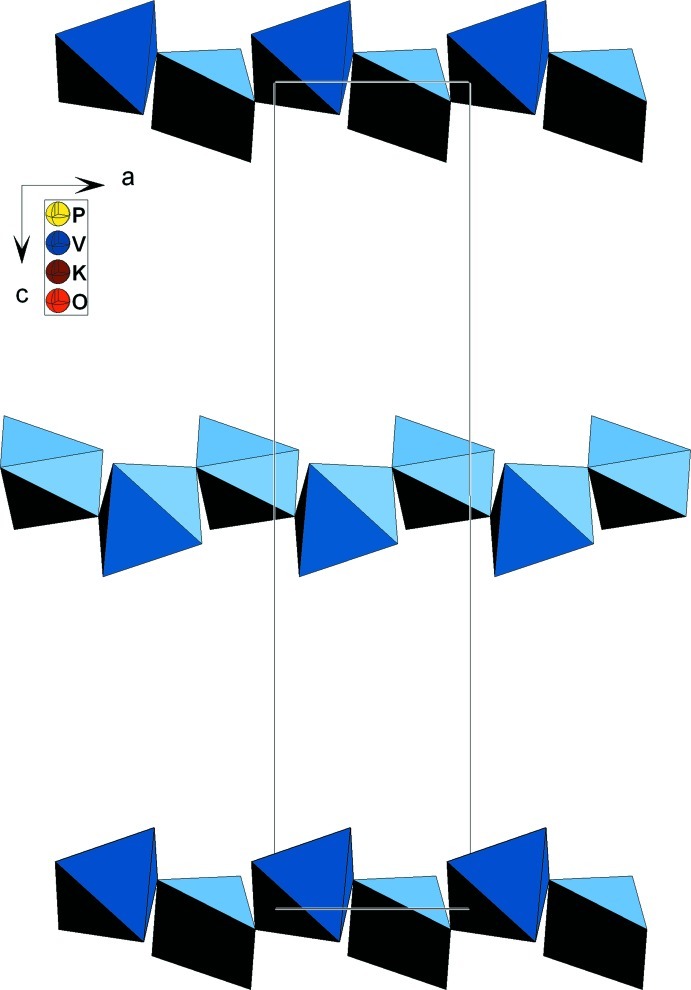
Representation of the second type of (V2O8)∞ chains, running along a.

**Fig. 4.**
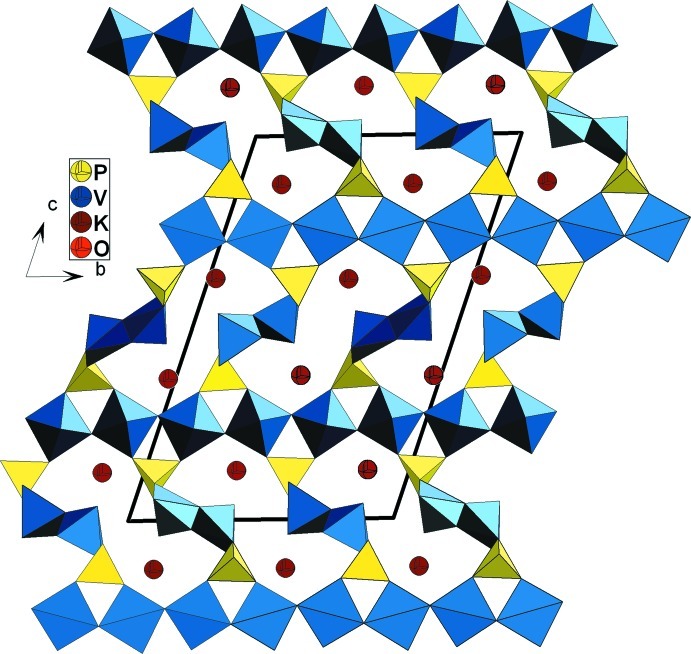
Projection of the structure of β-K(VO2)2(PO4) along a showing S shaped tunnels.

**Fig. 5.**
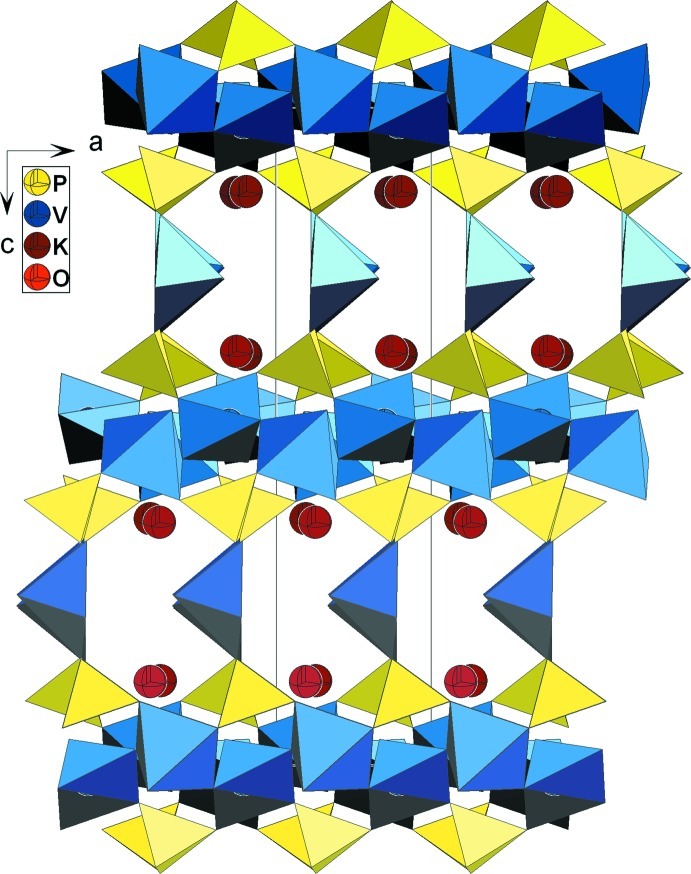
Projection of the structure of β-K(VO2)2(PO4) along b showing 'Pacman' shaped tunnels.

## supplementary crystallographic information

### Comment

Materials developed in the A—V—*X*—O systems (where A=Alkali, *X*= P or As) and having open mixed anionic frameworks (uni, bi, and three-dimensional) have interesting physical properties, especially catalytic (Pierini *et al.*, 2005) and ionic conductivity (Daidouh *et al.*, 1997). This has led to the synthesis, by a solid state reaction, of a new form of divanadium phosphate KV_2_PO_8_. The asymmetric unit, (Fig. 1) of the title compound, is built up from four double pyramidal units V_2_O_9_ (consisting of distorted vertices-sharing VO_5_ trigonal-bipyramids encountered in A_6_V_6_P_6_O_31_ (A= Rb and K (Benhamada *et al.*, 1991; Leclaire & Raveau, 2006)) interconnected by corner-sharing PO_4_ single tetrahedra. The junction of each kind of V_2_O_9_ unit by V–O–V bridges leads to two types of infinite chains with formula (V_2_O_8_)_∞_. The first kind is parallel to the *b* axis where two successive pairs of pyramids have their apical oxygen atoms pointing towards the same direction (Fig. 2a). The second type is parallel to the *a* axis (Fig. 3) and two successive pairs of pyramids have their apical oxygen atoms pointing towards opposite directions. The compound can be described as a connection between the (V_2_O_8_)_∞_ chains by vertex sharing of VO_5_ pentahedra and PO_4_ tetrahedra. Projections of the structure along the *a* and *b* axis show that the VO_5_ and PO_4_ polyhedra alternate along the *a* axis to form infinite chains (VPO_7_)_∞_ in which vanadium polyhedra share an oxygen with one neighboring chain, leading to double chains (V_2_P_2_O_12_)_∞_. Adjacent (V_2_O_8_)_∞_ chains running along *b* are connected by double chains of vanadyl phosphate *via* corner sharing. The structure is depicted in (Fig. 4) and (Fig. 5) and can be denoted as a three-dimensional framework with two types of tunnels running along *a* and *b* axis where the monovalent cations K^+^ are located.

The bond valence sums (BVS) calculations using the empirical formula of Brown (Brown & Altermatt, 1985) assuming cations bonds give the values: P1(5.045), P2(4.991), P3(5.013), P4(5.027), V1(5.106), V2(5.066), V3(5.028), V4(5.127), V5(5.056), V6(5.081), V7(5.087), V8(5.115), K1(1.029), K2(0.991), K3(1.031) and K4(1.004) which verify coordination geometries and oxidation states for each atom.

The comparison of our material with those found in the literature reveals the presence of infinite chains (VPO_7_)_∞_, (V_2_P_2_O_12_)_∞_ and (V_2_O_8_)_∞_ in the noncentrosymmetric compound with similar formulation α-KV_2_PO_8_ (Berrah *et al.*, 1999). However, two successive pairs of pyramids have their apical oxygen in a *trans*-position leading to the sequence "*cis*-*trans*-*trans*-*cis*" (Fig. 2b). This leads to eight-sided tunnels that are smaller than those encountered in our structure. (V_2_O_8_)_∞_ chains similar to those found in our phase where two successive pairs of pyramids have their apical oxygen atoms pointing towards the same direction have already been observed for the ammonium hydrogenophosphate α-NH_4_VO_2_PO_3_OH (Amoros & Le Bail, 1992). Materials K_3_V_3_As_2_O_14_ (Ezzine *et al.*, 2009) and A_2_VP_2_O_8_ (A = Rb, Cs (Lii & Wang, 1989) and Na, Rb (Daidouh *et al.*, 1998)) also contain chains (VXO_7_)_∞_ (*X* = As or P) whose linkage form infinite layers of type (V_2_As_2_O_14_)_∞_ and (VP_2_O_8_)_∞_. The junction of these chains along the three directions of the cell edges leads to three-dimensional structures with large tunnels for α-KV_2_PO_8_ and K_3_V_3_As_2_O_14_ compounds. As far as the series of A_2_VP_2_O_8_ (A= Alkali) compounds is concerned, they form by P–O–P bridges two-dimensional structures, characterized by the presence of interlayer space where the cations are located.

### Experimental

In order to obtain a new phosphate isotypic with K_3_V_3_As_2_O_14_ (Ezzine *et al.*, 2009), KNO_3_ (Fluka, 60415), NH_4_VO_3_ (Riedel-De Haën, 12739) and NH_4_H_2_PO_4_ (Scharlau, AM0335) were first mixed in the molar ratio 3:3:2 and heated at 573 K to decompose the ammonium phosphate and the nitrate. In a second step, the resulting mixture was crushed then heated at 793 K for two days, cooled slowly (5°C/24 h) down to 743 K and finally quenched to room temperature. From the resulting product, well-shaped crystals of various sizes, of satisfactory quality for analysis by X-ray diffraction, were retrieved. A yellow ochre parallelpipedic crystal was chosen from the selection for the determination of cell parameters.

### Refinement

The electron density maximum and minimum in the remaining Fourier differences are acceptable and are located respectively at 0.88 Å from O25 and 0.73 Å from V2.

### Crystal data

**Table:**

K(VO_2_)_2_(PO_4_)	*Z* = 8
*M**_r_* = 299.95	*F*(000) = 1152
Triclinic, *P*1	*D*_x_ = 3.020 Mg m^−^^3^
Hall symbol: -P 1	Mo *K*α radiation, λ = 0.71073 Å
*a* = 4.7438 (8) Å	Cell parameters from 25 reflections
*b* = 13.889 (2) Å	θ = 10–16°
*c* = 21.201 (3) Å	µ = 3.71 mm^−^^1^
α = 70.89 (2)°	*T* = 298 K
β = 89.55 (3)°	Prism, yellow ochre
γ = 88.66 (3)°	0.28 × 0.18 × 0.12 mm
*V* = 1319.5 (4) Å^3^	

### Data collection

**Table:**

Enraf–Nonius CAD-4 diffractometer	4828 reflections with *I* > 2σ(*I*)
Radiation source: fine-focus sealed tube	*R*_int_ = 0.021
Graphite monochromator	θ_max_ = 27.0°, θ_min_ = 2.0°
ω/2θ scans	*h* = −6→1
Absorption correction: ψ scan (North *et al.*, 1968)	*k* = −17→17
*T*_min_ = 0.447, *T*_max_ = 0.668	*l* = −27→27
7567 measured reflections	2 standard reflections every 120 min
5673 independent reflections	intensity decay: 1.2%

### Refinement

**Table:**

Refinement on *F*^2^	Primary atom site location: structure-invariant direct methods
Least-squares matrix: full	Secondary atom site location: difference Fourier map
*R*[*F*^2^ > 2σ(*F*^2^)] = 0.030	*w* = 1/[σ^2^(*F*_o_^2^) + (0.045*P*)^2^ + 1.4475*P*] where *P* = (*F*_o_^2^ + 2*F*_c_^2^)/3
*wR*(*F*^2^) = 0.086	(Δ/σ)_max_ = 0.001
*S* = 1.08	Δρ_max_ = 0.80 e Å^−^^3^
5673 reflections	Δρ_min_ = −0.50 e Å^−^^3^
434 parameters	Extinction correction: *SHELXL97* (Sheldrick, 2008), Fc^*^=kFc[1+0.001xFc^2^λ^3^/sin(2θ)]^-1/4^
0 restraints	Extinction coefficient: 0.0177 (5)

### Special details

**Table:**

Geometry. All e.s.d.'s (except the e.s.d. in the dihedral angle between two l.s. planes) are estimated using the full covariance matrix. The cell e.s.d.'s are taken into account individually in the estimation of e.s.d.'s in distances, angles and torsion angles; correlations between e.s.d.'s in cell parameters are only used when they are defined by crystal symmetry. An approximate (isotropic) treatment of cell e.s.d.'s is used for estimating e.s.d.'s involving l.s. planes.
Refinement. Refinement of *F*^2^ against ALL reflections. The weighted *R*-factor *wR* and goodness of fit *S* are based on *F*^2^, conventional *R*-factors *R* are based on *F*, with *F* set to zero for negative *F*^2^. The threshold expression of *F*^2^ > σ(*F*^2^) is used only for calculating *R*-factors(gt) *etc*. and is not relevant to the choice of reflections for refinement. *R*-factors based on *F*^2^ are statistically about twice as large as those based on *F*, and *R*- factors based on ALL data will be even larger.

### Fractional atomic coordinates and isotropic or equivalent isotropic displacement parameters (Å^2^)

**Table:**

	*x*	*y*	*z*	*U*_iso_*/*U*_eq_	
P1	0.81894 (16)	0.82998 (6)	0.62044 (4)	0.00927 (16)	
P2	0.30877 (16)	0.66561 (6)	0.38286 (4)	0.00846 (16)	
P3	0.69764 (16)	0.95394 (6)	0.88110 (4)	0.00847 (16)	
P4	0.18695 (16)	0.55208 (6)	0.11864 (4)	0.00897 (16)	
V1	0.67895 (11)	0.62518 (4)	0.74349 (2)	0.01025 (12)	
V2	0.20740 (11)	0.84522 (4)	0.97612 (2)	0.00914 (12)	
V3	0.67812 (11)	0.86795 (4)	0.75923 (3)	0.01073 (12)	
V4	0.33078 (11)	0.63012 (4)	0.24341 (3)	0.01087 (12)	
V5	0.79712 (10)	0.68009 (4)	0.47752 (2)	0.00898 (12)	
V6	0.32962 (11)	0.87164 (4)	0.25925 (3)	0.01096 (12)	
V7	0.30088 (11)	0.82752 (4)	0.52064 (2)	0.00904 (12)	
V8	0.70261 (11)	0.65479 (4)	0.01947 (2)	0.00902 (12)	
K1	0.19194 (17)	0.53056 (6)	0.62537 (4)	0.02600 (18)	
K2	0.80918 (17)	0.84200 (7)	0.12683 (4)	0.02642 (18)	
K3	0.25658 (18)	0.65377 (7)	0.87178 (4)	0.02692 (19)	
K4	0.74706 (18)	0.97341 (6)	0.36973 (4)	0.02608 (19)	
O1	0.6015 (5)	0.78815 (16)	0.46573 (11)	0.0130 (4)	
O2	0.4024 (4)	0.74851 (16)	0.96467 (11)	0.0132 (4)	
O3	0.8822 (4)	0.93237 (16)	0.94288 (10)	0.0116 (4)	
O4	0.1257 (4)	0.85042 (17)	0.59847 (11)	0.0146 (4)	
O5	0.1243 (4)	0.62663 (16)	0.44482 (10)	0.0113 (4)	
O6	0.9010 (4)	0.75197 (16)	0.02434 (11)	0.0135 (4)	
O7	0.1026 (5)	0.72520 (17)	0.52599 (11)	0.0135 (4)	
O8	0.8785 (4)	0.55572 (16)	0.09744 (11)	0.0145 (5)	
O9	0.6199 (5)	0.65051 (17)	0.40334 (11)	0.0146 (4)	
O10	0.2347 (5)	0.77905 (16)	0.34598 (11)	0.0143 (5)	
O11	0.3322 (5)	0.86573 (18)	0.03997 (11)	0.0184 (5)	
O12	0.3868 (4)	0.94798 (16)	0.90171 (11)	0.0136 (4)	
O13	0.2543 (5)	0.93608 (16)	0.16436 (11)	0.0129 (4)	
O14	0.6717 (5)	0.59606 (18)	0.54165 (11)	0.0177 (5)	
O15	0.7832 (6)	0.71576 (17)	0.65851 (11)	0.0201 (5)	
O16	0.3734 (4)	0.57915 (17)	0.05664 (11)	0.0141 (4)	
O17	0.7492 (5)	0.89548 (16)	0.66473 (11)	0.0131 (4)	
O18	0.2579 (5)	0.59998 (16)	0.33811 (11)	0.0126 (4)	
O19	0.2327 (6)	0.62756 (17)	0.15666 (11)	0.0196 (5)	
O20	0.1780 (5)	0.92122 (18)	0.46105 (12)	0.0195 (5)	
O21	0.8267 (5)	0.62017 (18)	0.96030 (12)	0.0184 (5)	
O22	0.7748 (5)	0.87717 (16)	0.84469 (11)	0.0143 (5)	
O23	0.6303 (5)	0.86329 (17)	0.55904 (11)	0.0143 (4)	
O24	0.2538 (5)	0.44138 (16)	0.16220 (11)	0.0131 (4)	
O25	0.2320 (5)	0.98015 (16)	0.27381 (11)	0.0174 (5)	
O26	0.7750 (5)	0.74426 (16)	0.77524 (11)	0.0163 (5)	
O27	0.2304 (5)	0.75295 (16)	0.22901 (11)	0.0175 (5)	
O28	0.7767 (5)	0.51742 (16)	0.72784 (11)	0.0166 (5)	
O29	0.3424 (5)	0.62895 (19)	0.74222 (12)	0.0219 (5)	
O30	0.6654 (5)	0.8693 (2)	0.25991 (13)	0.0236 (5)	
O31	0.3413 (5)	0.86967 (19)	0.76068 (12)	0.0215 (5)	
O32	0.6666 (5)	0.6285 (2)	0.24292 (13)	0.0231 (5)	

### Atomic displacement parameters (Å^2^)

**Table:**

	*U*^11^	*U*^22^	*U*^33^	*U*^12^	*U*^13^	*U*^23^
P1	0.0107 (4)	0.0107 (3)	0.0072 (3)	−0.0017 (3)	0.0015 (3)	−0.0039 (3)
P2	0.0090 (4)	0.0101 (3)	0.0066 (3)	−0.0001 (3)	−0.0001 (3)	−0.0032 (3)
P3	0.0086 (4)	0.0090 (3)	0.0070 (3)	−0.0001 (3)	0.0002 (3)	−0.0016 (3)
P4	0.0105 (4)	0.0087 (3)	0.0069 (3)	−0.0003 (3)	−0.0015 (3)	−0.0013 (3)
V1	0.0148 (3)	0.0087 (2)	0.0071 (2)	−0.00055 (19)	−0.00053 (19)	−0.00236 (19)
V2	0.0078 (2)	0.0107 (2)	0.0082 (2)	0.00032 (18)	−0.00125 (18)	−0.00212 (19)
V3	0.0153 (3)	0.0088 (2)	0.0081 (2)	−0.00139 (19)	0.00208 (19)	−0.00273 (19)
V4	0.0157 (3)	0.0089 (2)	0.0081 (2)	−0.00129 (19)	−0.0007 (2)	−0.00267 (19)
V5	0.0072 (2)	0.0115 (2)	0.0083 (2)	−0.00063 (18)	0.00118 (18)	−0.00344 (19)
V6	0.0161 (3)	0.0088 (2)	0.0082 (2)	−0.00082 (19)	−0.0007 (2)	−0.00290 (19)
V7	0.0077 (2)	0.0114 (2)	0.0083 (2)	0.00012 (18)	−0.00159 (18)	−0.00353 (19)
V8	0.0081 (2)	0.0101 (2)	0.0083 (2)	−0.00074 (18)	0.00148 (19)	−0.00222 (19)
K1	0.0215 (4)	0.0238 (4)	0.0267 (4)	−0.0012 (3)	0.0034 (3)	−0.0002 (3)
K2	0.0228 (4)	0.0352 (4)	0.0265 (4)	0.0008 (3)	−0.0044 (3)	−0.0173 (4)
K3	0.0252 (4)	0.0351 (4)	0.0268 (4)	−0.0088 (3)	0.0098 (3)	−0.0186 (4)
K4	0.0262 (4)	0.0217 (4)	0.0252 (4)	−0.0035 (3)	−0.0088 (3)	−0.0003 (3)
O1	0.0112 (10)	0.0162 (11)	0.0130 (10)	0.0018 (8)	−0.0012 (8)	−0.0069 (9)
O2	0.0106 (10)	0.0134 (10)	0.0139 (11)	−0.0001 (8)	0.0009 (8)	−0.0023 (8)
O3	0.0109 (10)	0.0149 (10)	0.0085 (10)	0.0027 (8)	−0.0021 (8)	−0.0032 (8)
O4	0.0077 (10)	0.0244 (12)	0.0149 (11)	−0.0013 (9)	0.0013 (8)	−0.0110 (9)
O5	0.0090 (10)	0.0150 (10)	0.0085 (10)	0.0021 (8)	0.0012 (8)	−0.0024 (8)
O6	0.0092 (10)	0.0120 (10)	0.0169 (11)	−0.0007 (8)	−0.0019 (9)	−0.0014 (8)
O7	0.0099 (10)	0.0176 (11)	0.0160 (11)	−0.0025 (8)	0.0016 (9)	−0.0094 (9)
O8	0.0083 (10)	0.0172 (11)	0.0142 (11)	−0.0006 (8)	0.0006 (8)	0.0001 (9)
O9	0.0095 (11)	0.0224 (11)	0.0156 (11)	0.0013 (9)	−0.0015 (9)	−0.0114 (9)
O10	0.0217 (12)	0.0102 (10)	0.0103 (10)	0.0013 (9)	−0.0003 (9)	−0.0025 (8)
O11	0.0184 (12)	0.0234 (12)	0.0137 (11)	−0.0025 (9)	−0.0029 (9)	−0.0063 (9)
O12	0.0082 (10)	0.0146 (10)	0.0137 (11)	0.0003 (8)	−0.0015 (8)	0.0011 (8)
O13	0.0190 (12)	0.0105 (10)	0.0087 (10)	−0.0037 (8)	0.0014 (9)	−0.0022 (8)
O14	0.0149 (11)	0.0218 (12)	0.0135 (11)	−0.0046 (9)	0.0032 (9)	−0.0015 (9)
O15	0.0389 (15)	0.0112 (11)	0.0105 (11)	−0.0044 (10)	0.0079 (10)	−0.0040 (9)
O16	0.0095 (10)	0.0193 (11)	0.0122 (10)	−0.0033 (8)	0.0012 (8)	−0.0030 (9)
O17	0.0187 (12)	0.0112 (10)	0.0097 (10)	0.0000 (8)	0.0032 (9)	−0.0041 (8)
O18	0.0176 (11)	0.0128 (10)	0.0078 (10)	−0.0019 (8)	−0.0010 (8)	−0.0037 (8)
O19	0.0354 (14)	0.0116 (11)	0.0119 (11)	−0.0016 (10)	−0.0066 (10)	−0.0038 (9)
O20	0.0195 (12)	0.0199 (12)	0.0161 (12)	0.0051 (9)	−0.0034 (10)	−0.0021 (9)
O21	0.0183 (12)	0.0234 (12)	0.0150 (11)	0.0029 (9)	0.0039 (9)	−0.0085 (9)
O22	0.0214 (12)	0.0105 (10)	0.0113 (11)	0.0003 (9)	0.0010 (9)	−0.0041 (8)
O23	0.0105 (11)	0.0223 (11)	0.0117 (10)	−0.0019 (9)	−0.0019 (8)	−0.0076 (9)
O24	0.0197 (12)	0.0095 (10)	0.0090 (10)	0.0015 (8)	−0.0022 (9)	−0.0017 (8)
O25	0.0343 (14)	0.0103 (10)	0.0075 (10)	−0.0020 (9)	0.0023 (9)	−0.0028 (8)
O26	0.0294 (13)	0.0107 (10)	0.0086 (10)	0.0004 (9)	−0.0008 (9)	−0.0029 (8)
O27	0.0334 (14)	0.0109 (10)	0.0075 (10)	−0.0016 (9)	−0.0031 (10)	−0.0017 (8)
O28	0.0317 (13)	0.0099 (10)	0.0081 (10)	−0.0020 (9)	0.0023 (9)	−0.0027 (8)
O29	0.0166 (12)	0.0270 (13)	0.0201 (13)	0.0027 (10)	−0.0033 (10)	−0.0049 (10)
O30	0.0173 (13)	0.0282 (13)	0.0248 (13)	0.0003 (10)	−0.0031 (10)	−0.0082 (11)
O31	0.0165 (12)	0.0269 (13)	0.0213 (13)	−0.0015 (10)	0.0030 (10)	−0.0080 (10)
O32	0.0155 (12)	0.0304 (14)	0.0243 (13)	−0.0037 (10)	0.0018 (10)	−0.0098 (11)

### Geometric parameters (Å, º)

**Table:**

P1—O23	1.521 (3)	V7—O7	1.695 (2)
P1—O4^i^	1.529 (2)	V7—O23	1.917 (2)
P1—O17	1.536 (2)	V7—O4	1.955 (2)
P1—O15	1.539 (2)	V7—O1	2.010 (3)
P2—O5	1.524 (2)	V8—O21^vii^	1.588 (2)
P2—O9	1.532 (2)	V8—O6	1.694 (2)
P2—O18	1.539 (2)	V8—O16	1.918 (3)
P2—O10	1.546 (2)	V8—O8	1.950 (3)
P3—O3	1.523 (2)	V8—O2^vii^	2.005 (3)
P3—O12	1.531 (2)	K1—O18^viii^	2.771 (3)
P3—O13^ii^	1.537 (2)	K1—O14	2.852 (3)
P3—O22	1.544 (2)	K1—O7	2.856 (3)
P4—O16	1.525 (3)	K1—O28^iii^	2.885 (3)
P4—O8^iii^	1.529 (2)	K1—O9^iv^	2.895 (3)
P4—O19	1.537 (2)	K1—O14^iii^	2.996 (3)
P4—O24	1.539 (2)	K1—O32^iv^	3.008 (4)
V1—O29	1.596 (2)	K1—O18^iv^	3.097 (3)
V1—O28	1.690 (2)	K1—O29	3.287 (3)
V1—O15	1.899 (3)	K2—O13^i^	2.763 (3)
V1—O24^iv^	1.934 (2)	K2—O6	2.866 (3)
V1—O26	2.041 (2)	K2—O11	2.873 (3)
V2—O11^v^	1.593 (2)	K2—O27^i^	2.901 (4)
V2—O2	1.694 (2)	K2—O12^ii^	2.910 (3)
V2—O3^iii^	1.927 (3)	K2—O11^i^	3.037 (3)
V2—O12	1.952 (3)	K2—O30	3.038 (3)
V2—O6^vi^	2.008 (3)	K2—O13	3.124 (3)
V3—O31	1.598 (2)	K2—O32	3.256 (4)
V3—O26	1.692 (2)	K3—O21^iii^	2.704 (3)
V3—O22	1.918 (2)	K3—O2	2.802 (3)
V3—O17	1.941 (2)	K3—O24^iv^	2.848 (3)
V3—O25^ii^	2.048 (2)	K3—O8^iv^	2.853 (3)
V4—O32	1.592 (2)	K3—O29	2.903 (3)
V4—O27	1.689 (2)	K3—O24^viii^	2.988 (3)
V4—O19	1.912 (2)	K3—O26^iii^	3.040 (4)
V4—O18	1.942 (2)	K3—O31	3.181 (4)
V4—O28^iv^	2.014 (2)	K3—O26	3.193 (4)
V5—O14	1.595 (3)	K3—O21	3.240 (3)
V5—O1	1.694 (2)	K4—O20^i^	2.744 (3)
V5—O5^i^	1.926 (2)	K4—O1	2.806 (3)
V5—O9	1.952 (2)	K4—O4^ii^	2.822 (3)
V5—O7^i^	2.009 (3)	K4—O17^ii^	2.889 (3)
V6—O30	1.593 (3)	K4—O31^ii^	2.933 (3)
V6—O25	1.688 (2)	K4—O17^ix^	2.977 (3)
V6—O10	1.924 (3)	K4—O25^i^	3.041 (3)
V6—O13	1.948 (2)	K4—O30	3.147 (3)
V6—O27	2.021 (2)	K4—O25	3.171 (3)
V7—O20	1.590 (3)	K4—O20	3.264 (4)
			
O23—P1—O4^i^	109.01 (14)	O22—V3—O25^ii^	83.48 (11)
O23—P1—O17	109.42 (14)	O17—V3—O25^ii^	76.99 (11)
O4^i^—P1—O17	106.61 (14)	O32—V4—O27	105.91 (15)
O23—P1—O15	110.17 (15)	O32—V4—O19	103.89 (15)
O4^i^—P1—O15	109.90 (17)	O27—V4—O19	95.54 (12)
O17—P1—O15	111.63 (13)	O32—V4—O18	100.53 (14)
O5—P2—O9	109.44 (14)	O27—V4—O18	90.26 (12)
O5—P2—O18	108.38 (13)	O19—V4—O18	152.23 (11)
O9—P2—O18	106.84 (14)	O32—V4—O28^iv^	105.13 (15)
O5—P2—O10	109.41 (14)	O27—V4—O28^iv^	148.24 (12)
O9—P2—O10	111.43 (16)	O19—V4—O28^iv^	83.39 (11)
O18—P2—O10	111.26 (13)	O18—V4—O28^iv^	77.61 (11)
O3—P3—O12	109.42 (13)	O14—V5—O1	106.78 (13)
O3—P3—O13^ii^	108.56 (14)	O14—V5—O5^i^	110.53 (13)
O12—P3—O13^ii^	106.81 (15)	O1—V5—O5^i^	142.54 (11)
O3—P3—O22	109.33 (14)	O14—V5—O9	103.24 (12)
O12—P3—O22	111.71 (15)	O1—V5—O9	93.29 (12)
O13^ii^—P3—O22	110.93 (13)	O5^i^—V5—O9	81.56 (10)
O16—P4—O8^iii^	109.06 (13)	O14—V5—O7^i^	96.23 (12)
O16—P4—O19	109.92 (14)	O1—V5—O7^i^	93.05 (11)
O8^iii^—P4—O19	110.26 (15)	O5^i^—V5—O7^i^	79.85 (10)
O16—P4—O24	108.94 (14)	O9—V5—O7^i^	156.79 (10)
O8^iii^—P4—O24	106.65 (15)	O30—V6—O25	105.48 (15)
O19—P4—O24	111.92 (13)	O30—V6—O10	103.21 (15)
O29—V1—O28	105.95 (15)	O25—V6—O10	96.97 (11)
O29—V1—O15	104.16 (15)	O30—V6—O13	101.01 (15)
O28—V1—O15	95.75 (11)	O25—V6—O13	90.43 (12)
O29—V1—O24^iv^	100.43 (15)	O10—V6—O13	151.70 (10)
O28—V1—O24^iv^	90.45 (11)	O30—V6—O27	103.95 (14)
O15—V1—O24^iv^	151.84 (11)	O25—V6—O27	149.67 (12)
O29—V1—O26	102.98 (14)	O10—V6—O27	83.24 (10)
O28—V1—O26	150.24 (12)	O13—V6—O27	76.87 (10)
O15—V1—O26	83.72 (10)	O20—V7—O7	107.46 (13)
O24^iv^—V1—O26	77.53 (10)	O20—V7—O23	111.76 (13)
O11^v^—V2—O2	106.92 (13)	O7—V7—O23	140.64 (12)
O11^v^—V2—O3^iii^	110.30 (13)	O20—V7—O4	101.96 (12)
O2—V2—O3^iii^	142.66 (11)	O7—V7—O4	94.01 (12)
O11^v^—V2—O12	103.25 (12)	O23—V7—O4	81.21 (10)
O2—V2—O12	93.04 (11)	O20—V7—O1	95.37 (13)
O3^iii^—V2—O12	81.45 (11)	O7—V7—O1	93.37 (11)
O11^v^—V2—O6^vi^	96.50 (12)	O23—V7—O1	80.05 (10)
O2—V2—O6^vi^	92.90 (11)	O4—V7—O1	158.15 (10)
O3^iii^—V2—O6^vi^	80.19 (10)	O21^vii^—V8—O6	107.21 (13)
O12—V2—O6^vi^	156.70 (10)	O21^vii^—V8—O16	110.63 (12)
O31—V3—O26	105.43 (15)	O6—V8—O16	142.02 (11)
O31—V3—O22	102.93 (14)	O21^vii^—V8—O8	102.02 (12)
O26—V3—O22	97.24 (12)	O6—V8—O8	93.61 (11)
O31—V3—O17	101.01 (14)	O16—V8—O8	81.75 (11)
O26—V3—O17	90.49 (12)	O21^vii^—V8—O2^vii^	95.70 (12)
O22—V3—O17	151.81 (10)	O6—V8—O2^vii^	93.36 (10)
O31—V3—O25^ii^	102.47 (15)	O16—V8—O2^vii^	79.98 (10)
O26—V3—O25^ii^	151.14 (12)	O8—V8—O2^vii^	158.08 (10)

Symmetry codes: (i) *x*+1, *y*, *z*; (ii) −*x*+1, −*y*+2, −*z*+1; (iii) *x*−1, *y*, *z*; (iv) −*x*+1, −*y*+1, −*z*+1; (v) *x*, *y*, *z*+1; (vi) *x*−1, *y*, *z*+1; (vii) *x*, *y*, *z*−1; (viii) −*x*, −*y*+1, −*z*+1; (ix) −*x*+2, −*y*+2, −*z*+1.
